# Integrin-Linked Kinase Is a Novel Therapeutic Target in Ovarian Cancer

**DOI:** 10.3390/jpm10040246

**Published:** 2020-11-26

**Authors:** Michael A. Ulm, Tiffany M. Redfern, Ben R. Wilson, Suriyan Ponnusamy, Sarah Asemota, Patrick W. Blackburn, Yinan Wang, Adam C. ElNaggar, Ramesh Narayanan

**Affiliations:** 1 Division of Gynecologic Oncology, West Cancer Center and Research Institute, Memphis, TN 38138, USA; mulm@westclinic.com (M.A.U.); tredfern@westclinic.com (T.M.R.); bwilson@westclinic.com (B.R.W.); pblackburn@westclinic.com (P.W.B.); aelnaggar@westclinic.com (A.C.E.); 2 Department of Medicine, University of Tennessee Health Science Center, Memphis, TN 38163, USA; tponnusa@uthsc.edu (S.P.); vqp741@uthsc.edu (S.A.); 3 Department of Pathology, University of Tennessee Health Science Center, Memphis, TN 38163, USA; ywang127@uthsc.edu

**Keywords:** integrin-linked kinase (ILK), ovarian cancer, sgRNA, gene expression, microarray, xenograft

## Abstract

Objective: The objective of this study is to identify and validate novel therapeutic target(s) in ovarian cancer. Background: Development of targeted therapeutics in ovarian cancer has been limited by molecular heterogeneity. Although gene expression datasets are available, most of them lack appropriate pair-matched controls to define the alterations that result in the transformation of normal ovarian cells to cancerous cells. Methods: We used microarray to compare the gene expression of treatment-naïve ovarian cancer tissue samples to pair-matched normal adjacent ovarian tissue from 24 patients. Ingenuity Pathway Analysis (IPA) was used to identify target pathways for further analysis. Integrin-linked kinase (ILK) expression in SKOV3 and OV90 cells was determined using Western blot. ILK was knocked down using CRISPR/Cas9 constructs. Subcutaneous xenograft study to determine the effect of ILK knockdown on tumor growth was performed in NOD SCID gamma mice. Results: Significant upregulation of the ILK pathway was identified in 22 of the 24 cancer specimens, identifying it as a potential player that could contribute to the transformation of normal ovarian cells to cancerous cells. Knockdown of ILK in SKOV3 cells resulted in decreased cell proliferation and tumor growth, and inhibition of downstream kinase, AKT (protein kinase B). These results were further validated using an ILK-1 chemical inhibitor, compound 22. Conclusion: Our initial findings validate ILK as a potential therapeutic target for molecular inhibition in ovarian cancer, which warrants further investigation.

**Highlights:**

- Integrin-linked kinase (ILK) is upregulated in ovarian cancer specimens relative to normal adjacent tissue specimens.
- ILK siRNA and small-molecule ILK-selective inhibitor (compound 22) inhibited the proliferation of ovarian cancer cells.
- ILK sgRNA lentiviral knockdown in SKOV3 cells resulted in slower tumor growth in NSG mice.
- ILK warrants further investigation as a potential therapeutic target for the treatment of ovarian cancer.

## 1. Introduction

A hallmark of ovarian cancer is the aggressive and silent nature of metastasis, predominantly through direct extension, into the peritoneal cavity [1]. Metastases are most commonly found within the omentum, the peritoneum, the diaphragm, and bowel surfaces [1,2]. This intraperitoneal dissemination requires detachment, or exfoliation, from the primary tumor on the ovary or fallopian tube [1]. This disruption of integrin–extracellular matrix interactions in normal epithelial cells induces apoptosis [3]. Thus, reduced sensitivity appears to be a hallmark of oncogenic transformation.

It is important to understand epithelial ovarian cancer at the molecular level to determine the underlying causes for its aggressiveness and heterogeneity. Several genome-wide expression studies have been conducted in epithelial ovarian cancer to determine the mechanism for the aggressive phenotype and to identify therapeutic targets [4,5,6].

Integrin-linked kinase (ILK), a serine-threonine kinase, has multiple functions in cells, such as cell–extracellular matrix interactions, cell cycle, apoptosis, cell proliferation, and cell motility [7,8,9]. Upregulation of ILK is frequently observed in cancer tissues compared to corresponding normal tissues [10]. Inhibition of ILK has been demonstrated to suppress activation of protein kinase Akt, inducing cell cycle arrest and apoptosis in prostate cancer [11] and colon cancer [12]. ILK is coexpressed with and activates the pro-metastatic enzyme membrane type 1 matrix metalloproteinase (MT1-MMP) in epithelial ovarian cancer cell lines. Downregulation of ILK using siRNA knockdown results in reduced adhesion to and invasion of collagen gels and organotypic meso-mimetic cultures, suggesting that ILK is integral to the development of metastatic disease in ovarian cancer [13].

In this study, we compared differential gene expression of high-grade, treatment-naïve ovarian cancer tissue samples to pair-matched adjacent benign ovarian tissue specimens to identify pathway(s) that are enriched in ovarian cancer tissues compared to adjoining normal ovarian cells. The ILK pathway was identified as the primary pathway that was enriched in cancer tissues compared to adjacent normal tissues. Downregulation of ILK using sgRNA resulted in reduced cell proliferation and tumor growth, confirming ILK as a valid therapeutic target.

## 2. Materials and Methods

*Reagents.* TaqMan PCR primers and fluorescent probes, master mixes, and Cells-to-Ct reagents were obtained from Life Technologies (Carlsbad, CA, USA). Cell culture medium and fetal bovine serum were purchased from Fisher Scientific (Waltham, MA, USA). Glyceraldehyde 3-phosphate dehydrogenase (GAPDH) antibody was purchased from Sigma (St. Louis, MO, USA). All other reagents used were of analytical grade. siRNA (Dharmacon Accell on-target plus pool) was ordered from Fisher Scientific. ILK sgRNA CRISPR/Cas9 all-in-one lentiviral vector set (cat. No. K2822105) was procured from Applied Biological Materials Inc. (Richmond, BC, Canada). ILK and pAKT antibodies were procured from Cell Signaling (Danvers, MA, USA). Compound 22 was procured from Millipore (Burlington, MA, USA).

*Patient specimen collection.* Patient specimens were collected under a University of Tennessee Health Science Center (UTHSC) Institutional Review Board (IRB) approved protocol (14-03113-XP). The Cooperative Human Tissue Network (CHTN) database was searched for ovarian cancer specimens that satisfied the following criteria.

- Histological grade 2 or 3.
- Tumors with adjoining normal ovarian tissue available.
- Treatment naïve.
- Snap frozen to facilitate isolation of high-quality RNA appropriate for microarray.

Out of the more than 10,000 ovarian tumors available in the CHTN database only 24 matched all of these criteria. Three out of the 24 tumors had histological grade less than 3, while the rest are of grade 3. In addition, five specimens were of non-serous epithelial carcinoma type. Histological analysis determined that the tumors contain between 70 and 100% cancer cells.

*Microarray.* RNA from tumors and benign ovarian specimens was extracted using the Qiagen RNA isolation kit (Qiagen, Hilden, Germany). Quantity was verified using nanodrop and the quality of RNA was verified using the Agilent bioanalyzer. Total RNA (200 ng/sample) from each sample was amplified and labeled using WT Plus Kit from Affymetrix and processed according to Affymetrix protocol. The arrays (Human ST2.0, Affymetrix, Santa Clara, CA, USA) were washed and stained on Affymetrix Fluidics station 450 and scanned on an Affymetrix GCS 3000 scanner. Data from microarrays were normalized using Affymetrix Expression Console. Mean, standard deviation, and variance were calculated across the groups. Fold change from vehicle-treated samples was calculated, and a fold change of 1.5 was used as the cutoff. Pair-wise Student’s t-tests were used to determine significance using the cutoff of a *p* value < 0.05. The false discovery rate (FDR) was calculated using the Benjamini and Hochberg method, and a cutoff for FDR of <0.05 was used to create a significant differential expression list. The gene candidate list was loaded to Ingenuity Pathway Analysis and gene set enrichment analysis (GSEA) was performed for further discovery. Microarray experiments were performed at the UTHSC Molecular Resources Center (MRC), and data analysis was performed by the UTHSC Molecular Bioinformatics core facility. Pathway analysis was also performed using pathway analysis software (Advaita bioinformatics, Ann Arbor, MI). Km plotter was used to obtain Kaplan–Meier plot for ovarian cancer from the Cancer Genome Atlas (TCGA) database [14,15].

*Real-time polymerase chain reaction (PCR).* Real-time PCR was performed as described previously [16,17]. For RNA isolation and real-time PCR in cells, cells were plated in 96 well plates. RNA was isolated using cells to ct kit and real-time PCR was performed using TaqMan primers and probes on an ABI 7900 real-time PCR machine. RNA from tissues were isolated using RNA isolation kit from Qiagen as described above under the microarray analysis. Total RNA was reverse transcribed into cDNA using reverse transcription kit and real-time PCR was performed for the specified genes using TaqMan real-time PCR primers and probes.

*Cell culture.* COS7, OV90 and SKOV3 cells were obtained from American Type Culture Collection (ATCC, Manassas, VA, USA). The cells were cultured in accordance with the ATCC recommendations. Respective medium was supplemented with 10% FBS and 1% penicillin-streptomycin. Cells were passaged every third day.

*Growth assay.* Cells were harvested by trypsinization, counted using a hemocytometer, and plated at 1000 cells per well on 96 well tissue culture plates in quadruplicates. Photomicrographs were taken every four hours using an INCUCYTE live cell imager (Essen Biosciences, AnnArbor, MI, USA) and confluence of the cultures was measured using INCUCYTE software (Essen Biosciences, Ann Arbor, MI, USA) over 72 and 144 h in culture. Simultaneously, cells were plated in 96 well plates. After the indicated period, sulforhodamine blue (SRB) assay was performed to measure the viable cells.

*Protein extraction and Western blot.* Cells for protein extraction were plated in 60 mm dishes in growth medium. Protein was extracted from tumors and cells as indicated before [16,17]. Protein samples were fractionated on a SDS-PAGE and Western blot was performed with the respective antibodies.

*siRNA transfection.* A titration starting from 50 nM of accell on-target plus pool siRNA was transfected into the cells using Dharmafect transfection reagent (Dharmacon, Lafayette, CO, USA). Twenty-four hours after transfection, medium was replaced, and the cells were allowed to recover. Efficiency of knockdown was evaluated three and six days after knockdown. GAPDH and scrambled siRNA were used as transfection controls.

*CRISPR/Cas9 Lentiviral ILK sgRNA knockdown*. Lentivirus carrying ILK sgRNAs (three different CRISPR/Cas9 clones) was produced by packaging in 293FT cells as published previously [18]. SKOV3 cells were plated in 6 well plates at 60% confluence 24 h before viral infection. Twenty-four hours after plating, wells were infected with 1 mL of virus suspension diluted in complete medium with Polybrene to a final concentration of 5–8 μg/mL. Cells were incubated for 48 h and then fed with fresh complete medium without Polybrene. Stable pools of ILK–KO cells were selected with 5 μg/mL puromycin treatment every 3–4 days until drug-resistant colonies were available.

*Tumor xenograft experiments.* All animal protocols were approved by the UTHSC Institutional Animal Care and Use (IACUC) research committee. Cells (5 million) were implanted subcutaneously in NOD SCID Gamma (NSG) mice. Tumor volume (length * width * width * 0.532) was measured three times weekly. Tumors were collected at sacrifice and stored for further processing. Microarray with Clariom D arrays was performed in the tumor specimens as indicated above. Tumor specimens collected in 10% neutral buffered formalin were sectioned and the sections were stained for hematoxylin and eosin (H&E) and the proliferation marker ki67.

*Statistics.* Statistical analysis was performed using GraphPad prism software (La Jolla, CA, USA). Experiments containing two groups were analyzed by simple t-test, while those containing more than two groups were analyzed by one-way analysis of variance (ANOVA) followed by Tukey’s post-hoc test. All in vitro experiments were performed at least in triplicate. Data are represented as the mean ± S.E. Significance is expressed as * *p* < 0.05, ** *p* < 0.01, and *** *p* < 0.001.

## 3. Results

**Figure 1:**
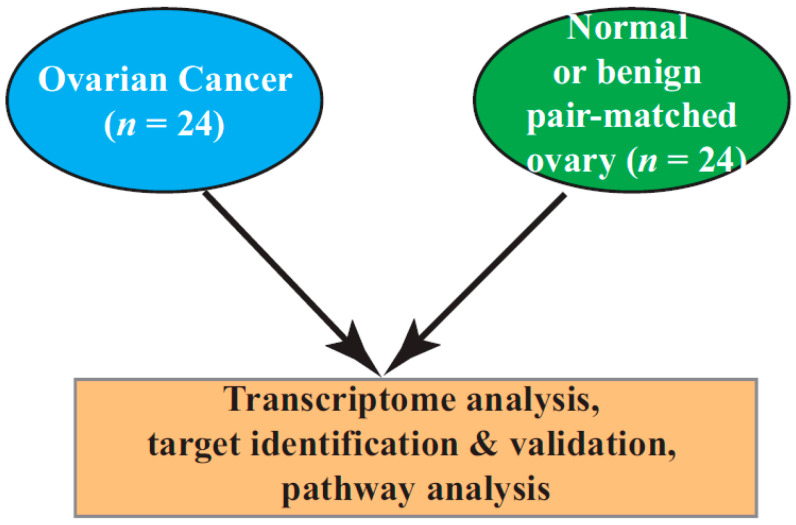
Experiment design. Ovarian cancer specimens (mostly histological grade 3) and adjacent normal tissue specimens (*n* = 24) were used in gene expression microarray experiments and pathway analyses.

**Figure 2:**
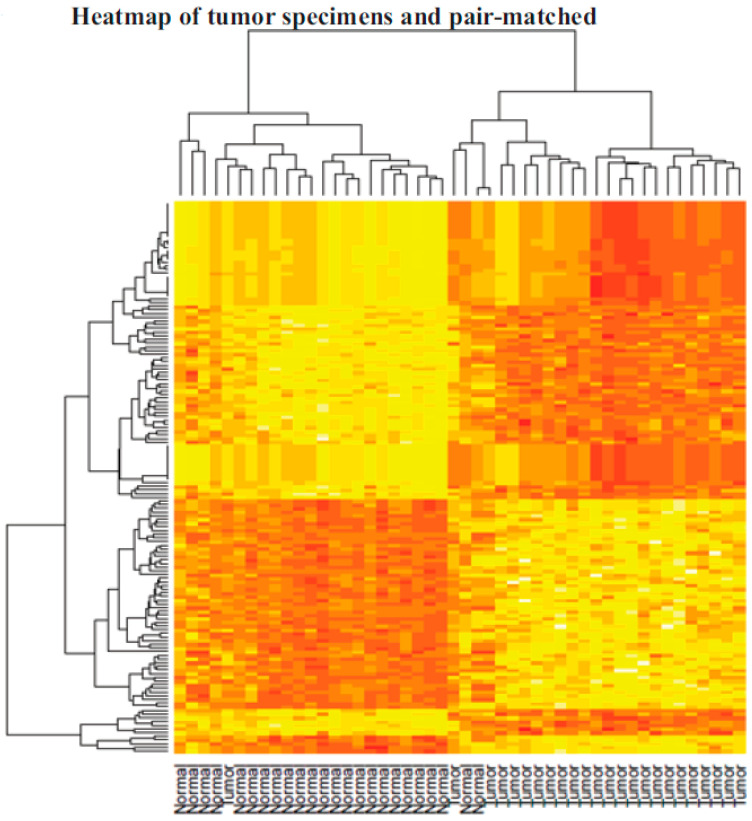
Pathway analysis. Heatmap of differentially regulated genes between tumor specimens and their respective pair-matched control specimens.

ILK is overexpressed in ovarian cancer relative to normal ovarian tissue: To identify reliable therapeutic targets for advanced ovarian cancer, high-grade treatment-naïve ovarian cancer specimens, and pair-matched adjacent normal ovarian tissues were obtained from the CHTN (Figure 1). The patient characteristics are provided in Table 1. Pair-matched normal ovarian specimens were allowed for the determination of alterations that took place in the ovarian cells that led to their transformation into cancerous cells. Out of almost 10,000 ovarian cancer specimens available in the CHTN, only 24 specimens met the criteria described above. Human Transcriptome Array (HT2.0) array was used to determine the genes, small non-coding RNAs, and pathways that were altered in cancer specimens compared to respective normal ovarian tissues. Using 1.5-fold up- or downregulation as a cutoff and an FDR of 0.05, we found that 994 genes and non-coding RNAs were differentially expressed in ovarian cancer specimens compared to pair-matched controls. The most upregulated genes included osteopontin (SPP1), ceruloplasmin (CP), desmoplasmin (DSP), epithelial splicing regulatory proteins (ESRP1), and cadherin (CDH1). The heatmap and unsupervised hierarchical clustering of statistically significant genes shows clustering of normal specimens (except for one normal specimen) to one side and the tumors to the other side (Figure 2).

The top 100 statistically significant genes were loaded into the Ingenuity Pathway Analysis (IPA) software for the identification of pathways and regulators that were enriched in this dataset (Table 2). The most enriched pathways identified by IPA are the integrin-linked pathway, tryptophan degradation X, putrescine degradation, insulin-like growth factor-1 signaling, and 14-3-3 sigma signaling. A significant enrichment of the ILK pathway was identified in 22 of the 24 cancer specimens, identifying this pathway as a potential player in the transformation of normal ovarian cells to cancerous cells (Table 2). The genes that represent the ILK pathway in the microarray included FOS, DSP, myosin 11 (MYH11), CDH1, mucin 1 (MUC1), and keratin (KRT18). While FOS and MYH11 were lower in the tumor specimens compared to their normal controls, the other genes were more prevalent in the tumor specimens. Interestingly, although c-FOS is a proto-oncogene, counterintuitively its expression was reduced in the cancer specimens. Evidences for c-FOS overexpression as promoting apoptosis and delaying ovarian cancer progression in preclinical models may be supported by the findings in these clinical specimens [19]. MYH11 expression has been shown to be downregulated in cancers, and its downregulation corresponds to poor prognosis and survival [20].

The top enriched upstream regulator pathway identified by IPA was the Wnt-1 inducible-signaling pathway (WISP1). The genes that encode the WISP1 pathway identified in the microarray dataset include cluster of differentiation (CD24), CDH1, DSP, KRT18, keratin 8 (KRT8), and MUC1. All of these genes were upregulated in cancer specimens compared to their respective normal controls. The WISP1 pathway is involved in cancer cell proliferation, invasion, and metastasis, and has been shown to be responsible for shorter patient survival [21]. Collectively, the genes enriched in the top canonical pathways and the upstream regulators indicate that the pathways responsible for tumor cell proliferation, metastasis, and invasion are altered to favor cancer growth and metastasis.

**Figure 3:**
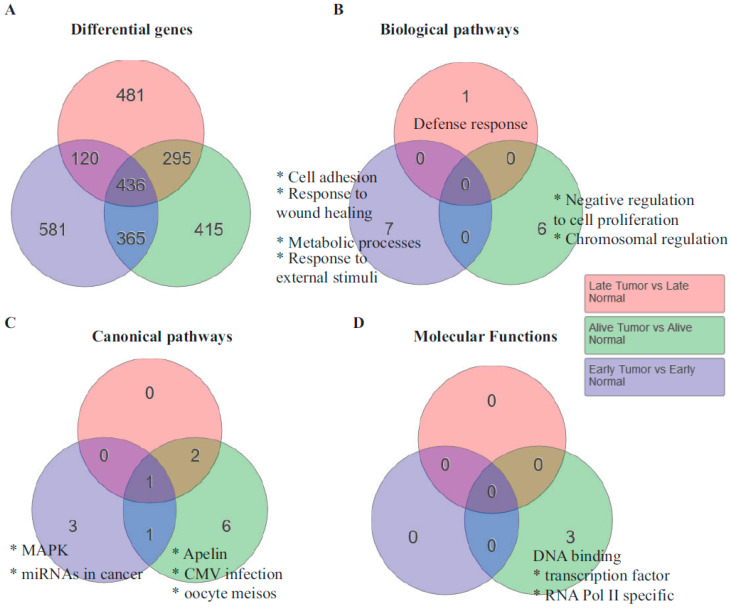
Pathway analysis of the differentially expressed genes with samples segregated based on survival. The numbers in the figures represent the number of genes (**A**) and the enriched pathways (**B**–**D**). The different pathways are represented in each Venn circle. Survival was defined as those patients who survived less than 30 months post-diagnosis (early), more than 30 months (late), and were alive at the time of data collection (alive).

Genes and pathways responsible for shorter survival: One of the interesting observations made in the specimens was that some patients survived longer, as much as six years since their first diagnosis, while several others had shorter survival from diagnosis (Table 1). To determine whether the genome-wide expression data provided any indication of the pathways that were contributing to shorter survival, we analyzed the genes based on survival. Unsupervised hierarchical clustering resulted in three subsets with regard to survival. They are patients who survived less than 30 months, those who survived greater than 30 months, and those who were alive at the time of the last data collection. Interestingly, 581 genes were statistically significant in patients who died less than 30 months after diagnosis. The biological pathways that were enriched in the early-death patients were cell adhesion, response to wound healing, and metabolic processes (Figure 3B). The most upregulated gene in the cell adhesion pathway was SPP1, a gene that encodes for osteopontin. Osteopontin that activates interleukin 17 (IL-17) has been shown to be upregulated in ovarian cancer and important for its metastasis [22].

The canonical pathway that was enriched in the patients who were alive at the time of sample collection (greater than six years) was the apelin pathway. Most of the genes in this pathway were downregulated. Considering that the apelin and its ligand apela are oncogenic [23], it is consistent that this pathway was downregulated in patients who survived the longest.

**Figure 4:**
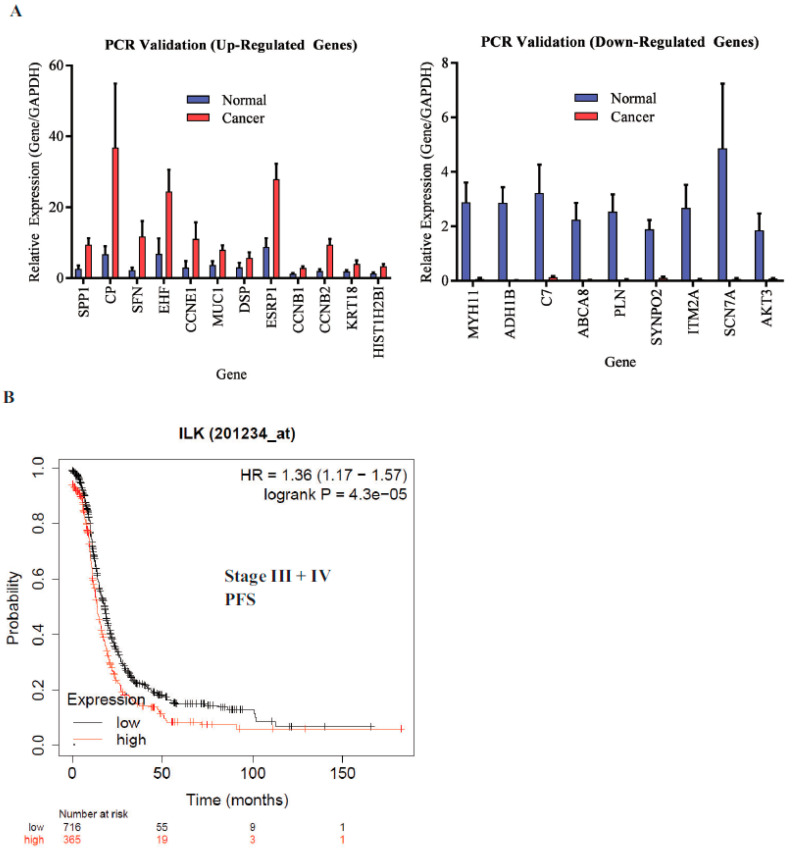
Validation of microarray data by real-time PCR. (**A**) Real-time PCR validation of a subset of genes identified by microarray (*n* = 5/gene). (**B**) Kaplan–Meier plot of the integrin-linked pathway (ILK-1) in the cancer genome atlas (TCGA).

ILK is associated with shorter progression-free survival (PFS): The microarray findings were validated by real-time PCR. All genes that were validated showed reproducible results (Figure 4A). ILK pathway genes DSP and MUC1 were included in the validation. We then determined the effect of ILK-1 high expression on stage III and IV ovarian cancer patients’ PFS in the TCGA database using Km plotter (Figure 4B). The probe range was between 32 and 3950 and a cutoff of 1335 was used to define high- vs. low-expression specimens. Ovarian cancer patients with high ILK expression had shorter PFS compared to patients with cancer that expressed lower ILK. The hazard ratio (HR) was 1.36 and the log-rank *p* was 0.000043. This validated the findings made in our study.

**Figure 5:**
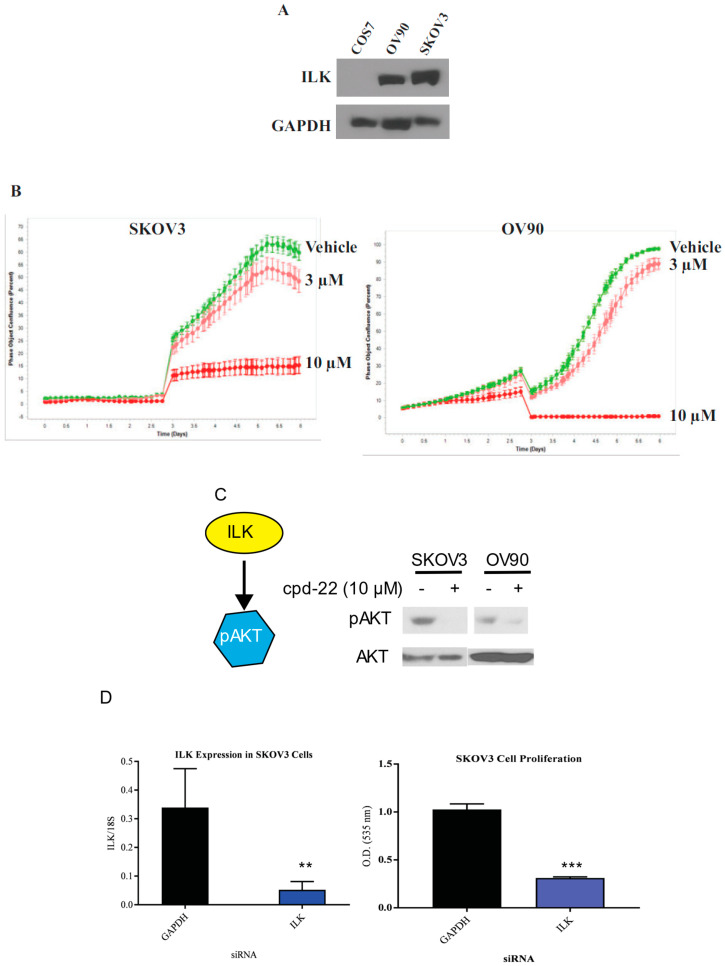
In vitro validation of the ILK pathway. (**A**) Protein expression of ILK-1 in two ovarian cancer cell lines, SKOV3 and OV90 and non-cancerous COS7 cells. Protein was extracted from the cells, fractionated on an SDS-PAGE, and Western blot for ILK-1 and GAPDH was performed. (**B**) INCUCYTE proliferation assay of SKOV3 and OV90 cell lines in the presence of vehicle (DMSO) or 3 or 10 μM ILK inhibitor compound 22. Images were obtained periodically for the indicated time-points. (**C**) Phosphorylation of AKT was inhibited by ILK-1 inhibitor compound 22. SKOV3 and OV90 cells were treated with compound 22 for 4 h. Cells were harvested, protein extracted, and Western blot with phospho-AKT and total AKT antibodies was performed. (**D**) ILK-1 siRNA inhibits SKOV3 cell proliferation. SKOV3 cells were transfected with ILK-1 or GAPDH siRNA. Six days after transfection (re-transfected after day 3) mRNA expression of ILK-1 and 18S (left) and cell proliferation (right) by sulforhodamine B (SRB) assay were measured (*n* = 3). ** *p* < 0.01; *** *p* < 0.001.

ILK is expressed in ovarian cancer cell lines: To determine whether ILK is expressed in ovarian cancer cell lines, Western blots in SKOV3 and OV90 ovarian cancer cell lines were performed and compared to non-cancerous COS7 cells. SKOV3 cells were chosen as they represented an established, well published serous or epithelial ovarian cancer cell line. OV90 cells were chosen as they are high-grade serous ovarian carcinoma and have an established track record in translational research. Figure 5A shows high expression of ILK in both ovarian cancer cell lines, while COS7 failed to express ILK-1 at detectable levels.

Compound 22 reduces the proliferation of SKOV3 and OV90 cell proliferation: Compound 22 (CP22), is a selective ILK inhibitor that was shown to be antiproliferative in prostate cancer [24]. To characterize the efficacy of CP22, we conducted proliferation studies in SKOV3 and OV90 cell lines in INCUCYTE. A prior dose response study was conducted to narrow down the doses of CP22 to 3 and 10 µM to be used in these experiments. Cells were incubated with DMSO or CP22 at 3 μM or 10 μM and imaged using INCUCYTE. CP22 effectively reduced the proliferation in a dose-dependent manner relative to vehicle control (Figure 5B).

Compound 22 facilitates dephosphorylation of Akt: In order to determine the efficacy of CP22 in ovarian cancer cell lines, Western blot for phosphorylated AKT was performed in SKOV3 and OV90 cells. AKT is a downstream target of ILK [11]. SKOV3 cells were incubated with DMSO or CP22 at 10 μM for 4 h and Western blot for AKT phosphorylation was performed. CP22 effectively inhibited the phosphorylation of AKT (Figure 5C).

siRNA knockdown of ILK reduced SKOV3 cell proliferation: To determine whether knockdown of ILK expression affects ovarian cancer cell proliferation, we transfected SKOV3 cells with ILK siRNA and measured the number of viable cells by SRB assay. Following transfection with siRNA directed against ILK, SKOV3 cells were incubated for 6 days. RT-PCR following siRNA directed knockdown of ILK showed a reduction in ILK expression in transfected cells compared to cells transfected with GAPDH (Figure 5D). A cell proliferation assay was performed following siRNA tranfection that showed a reduced cellular proliferation of SKOV3 cells following ILK siRNA transfection (Figure 5D). The results were comparable when scrambled, instead of GAPDH, siRNA was used as a control.

**Figure 6:**
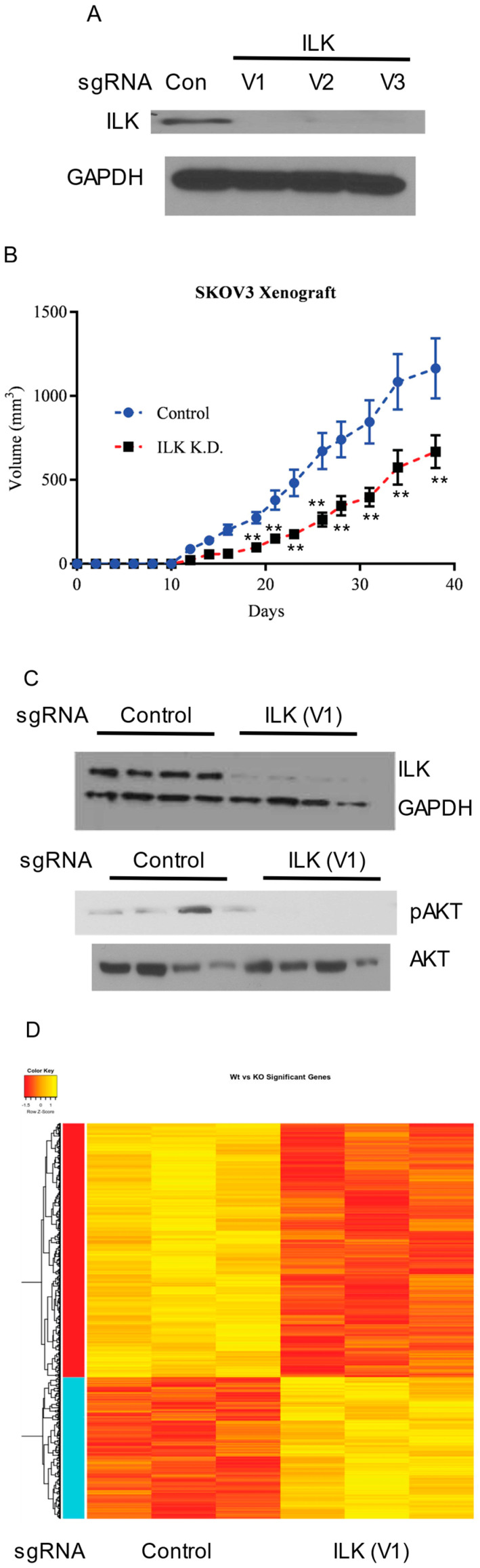

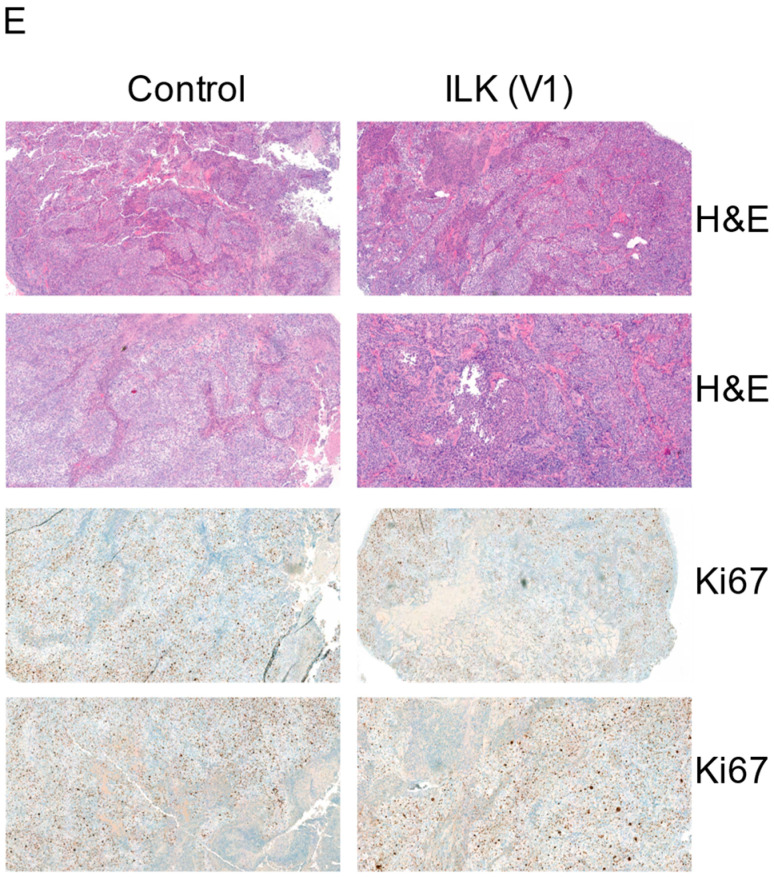
ILK-1 is important for SKOV3 tumor growth. (**A**) ILK-1 was knocked out using three CRISPR/Cas9 sgRNA. Western blot shows the knockout in SKOV3 cells. Control sgRNA was used in the vector-infected group. (**B**) Control and ILK-1 sgRNA knockdown cells (virus 1) were implanted in NSG mice (*n* = 15/group). Tumor uptake and growth were measured over the course of this study. Animals were sacrificed at the end of this study, tumors were isolated, and stored for further analysis. (**C**) Western blot in the tumors from animals described in panel B is provided. V1 corresponds to virus 1 (clone 1) of the three clones that were screened. (**D**) RNA was isolated from the tumors and the expression of genes in the vector or ILK-1 knockdown tumors (*n* = 3/group) was measured by microarray. (**E**) Representative H&E and ki67 staining of tumor sections. Statistically different genes between the two groups are represented as heatmap. ** *p* < 0.01.

ILK knockdown in SKOV3 cells results in tumor growth inhibition: To validate the findings obtained using a transient knockdown of ILK-1 using siRNAs, ILK was knocked down stably in SKOV3 cells using CRISPR/Cas9 sgRNA lentiviral constructs. Three sgRNAs to different regions of ILK were used to knockdown ILK. Lentivirus particles containing the CRISPR/Cas9 vectors were prepared and the cells were infected. Western blot showed that all three sgRNAs comparably knocked down ILK (Figure 6A). Control and ILK knockdown SKOV3 cells (clone 1 or virus 1) were implanted subcutaneously in NSG mice and tumor growth was monitored. Since the cells were not labeled with luciferase, subcutaneous, but not orthotopic, model was used to conduct the xenograft studies. ILK knockdown resulted in slower tumor uptake and growth (Figure 6B), confirming the observation made in vitro. Western blot with proteins extracted from tumor tissues confirmed the ILK knockdown and the inhibition of the downstream AKT phosphorylation (Figure 6C).

RNA was isolated from control and ILK knockdown tumors and microarray was performed to determine the effect of ILK knockdown on global gene expression. Knockdown of ILK resulted in alteration of 1301 genes with the hierarchical clustering demonstrating that ILK knockdown tissues clustered together (Figure 6D). The most significant pathway that was altered in ILK knockdown specimens was the ribosomal family of proteins (RPS) genes. Most of the RPS genes were downregulated in ILK knockdown specimens compared to control sgRNA specimens. In addition to the RPS pathway, metabolic pathways were also significantly downregulated in the ILK knockdown tumors.

The FFPE tumors were stained for H&E and ki67. As shown in Figure 6E, the tumor cell proliferation was lower in ILK sgRNA infected cells, matching the results observed with the tumor volume measurement. Representative images are included.

## 4. Discussion

Epithelial ovarian cancer is a heterologous disease in which the molecular and clinical phenotype can vary significantly between patients [25]. While platinum/taxane chemotherapy remains the standard basis for treatment of this difficult disease, the development of poly-ADP ribose inhibitors (PARPi) has ushered in a new era of molecular therapeutics for the treatment of ovarian cancer, taking advantage of deficiencies in the homologous recombination pathway. PARPi increased survival outcomes for patients with both somatic and germline mutations in BRCA and genes encoding the proteins involved in DNA repair by homologous recombination. For patients without somatic or germline homologous recombination mutations and for those whose cancer have become resistant to PARPi therapy, advances in targeted therapeutics are needed to improve survival of patients with advanced ovarian cancer [26]. Ongoing clinical trials in ovarian cancer are evaluating inhibitors of multiple molecular pathways such as PI3K/AKT, the mTOR pathway, angiogenesis, the MAPK pathway, and the HER/EGFR pathway [27,28]. Our preliminary results showing ILK inhibition in epithelial ovarian cancer highlight a novel pathway for the development of small-molecule inhibitors of this pathway for ovarian cancer treatment.

We discovered a significant upregulation of the ILK pathway through Ingenuity Pathway Analysis, identifying a novel target for molecular inhibition and validating work performed by Ahmed et al. in 2003 [29]. Similarly, upregulation of the ILK pathway is found in colorectal, breast, gastric and pancreatic carcinoma. The association between worse survival in patients with ovarian cancer who exhibit ILK upregulation is also true for these different cancers types as well [9]. In SKOV3 cells, inhibition of ILK using ILK-sgRNA results in upregulation of pro-apoptotic bax gene expression and downregulation of antiapoptotic genes in addition to reduced cell viability, similar to our results [8]. The downstream pathways that are significantly altered when ILK was knocked down include ribosome and metabolic pathways. Previous publications have demonstrated the importance of ribosomal proteins in ovarian cancer. Knockdown of RPS6 resulted in an inhibition of ovarian cancer cell proliferation and invasion [30]. Similarly, the metabolic pathways have been shown to be pivotal for the development of ovarian and other cancers [31]. Several of the ILK pathway genes such as DSP1, FOS, BMP2, and AKT3 that were altered between ovarian cancer and adjacent normal specimens in Figure 2 were altered in the knockdown dataset in Figure 6. These results collectively suggest that ILK is an important mediator of proliferation in ovarian cancer and attempts to deregulate this pathway will provide a bona fide therapeutic approach.

Furthermore, antisense oligonucleotide silencing of ILK expression has been shown to suppress tumor growth in nude mice xenografts [8]. ILK silencing has also been shown to reduce the expression of wnt ligands (wnt3a, wnt4, and wnt5a) and β-catenin in epithelial ovarian cancer cells [32]. Prior studies evaluating Compound 54 (CP54), a non-selective ILK inhibitor, failed to demonstrate activity in vivo due to its lack of specificity [33,34]. CP22 was developed as a highly selective inhibitor of ILK and was shown to have antiproliferative effects in both in vitro and in vivo experiments in prostate cancer [24]. Reyes-Gonzalez et al. showed that both ILK-siRNA and CP22 reduced cell growth, invasion ability and increased apoptosis in both cisplatin-sensitive and cisplatin-resistant ovarian cancer cell lines [35]. They also showed that high ILK expression in tumors from patients with ovarian cancer was associated with worse survival compared to patients with low ILK expression. Li et al. also showed that after transfection with ILK-antisense oligonucleotides, HO-8910 cells spent more time in in the G0/G1 phase, delayed tumor formation and decreased tumor growth compared to controls in xenograft models [36]. The HO-9810 cell line was originally thought to be derived from a 51 year old patient with serous ovarian cancer but was later found to be a derivation of the HeLa cell line, and is not an ovarian cancer model for validation of potential molecular targets for the treatment of ovarian cancer [37]. Our findings expanded on the findings of Reyes-Gonzalez et al. by validating that ILK silencing results in reduced in vivo tumor growth in a validate ovarian cancer cell line. The efficacy of CP22 in epithelial ovarian cancer cell lines and in vivo patient-derived xenografts validates the ability of small-molecule inhibitors to successfully target the ILK pathway in ovarian cancer and warrants further study. At the time of tissue collection, our goal was to obtain tissue from high-grade serous ovarian cancer specimens. The hypothesis regarding fallopian tube origin of high-grade serous ovarian carcinoma had not widely been accepted and thus fallopian tube was not considered normal matched paired tissue [38]. Although two specimens were ultimately found to be clear cell and transitional carcinoma in the analysis and recognize that these likely have different molecular signatures, they represented 2 out of the 24 specimens and we do not feel that their inclusion compromised our analysis. The normal adjacent tissue specimens contained epithelial, stroma and ovarian stem cells as well, not just epithelial tissue. ILK inhibition in ovarian cancer, however, has been shown to affect apoptotic, proliferative, and metastatic pathways.

The introduction of poly ADP-ribose (PARP) inhibition represented a paradigm shift in the traditional approach to ovarian cancer management by ushering in a new era where patient survival can be improved by delivering individualized therapeutics based upon germline and/or somatic testing [39]. Developing a similar understanding of response to ILK inhibition requires better understanding of the genetic alterations that predispose an individual’s tumor to respond. Murine models of patient-derived ovarian cancer tissue that have undergone next-generation sequencing are currently under development and will provide insight into the molecular profiles of tumors that respond to ILK inhibition. As more targeted therapeutics become available for patients with homologous recombination deficiency and mismatch repair deficiency, there is an increasing need for a large subset of patients who are not candidates for these targeted, life-saving therapies. ILK represents a potential pathway that may provide a promising alternative to PARPi or immunotherapy in patients who are homologous repair proficient and mismatch repair proficient.

## Figures and Tables

**Table 1:** Patient characteristics.

S. No.	Age	Histology	Hist. Grade	O.S. (Months after Diagnosis)
1	34	Endometrioid adenocarcinoma	1	8
2	41	Endometrioid adenocarcinoma	3	>72
3	57	Serous	3	0.3
4	65	Serous	3	>72
5	74	Serous	3	>72
6	75	Endometrioid adenocarcinoma	3	2
7	77	Serous	3	>72
8	51	Serous	3	48
9	64	Serous	3	24
10	49	Serous	3	7
11	80	Serous	3	8
12	68	Serous	3	>72
13	48	Serous	3	>60
14	52	Serous	3	7
15	54	Serous	3	5
16	55	Endometrioid adenocarcinoma	3	>60
17	66	Transitional cell carcinoma	3	14
18	37	Endometrioid adenocarcinoma	2	48
19	63	Clear cell carcinoma	N.D.	42
20	66	Endometrioid adenocarcinoma	2	36
21	56	Serous	3	42
22	65	Serous	3	30
23	72	Serous	3	24
24	64	Serous	3	>36

S. No.—sample number; O.S.—overall survival.

**Table 2:** Ingenuity Pathway Analysis.

Top Canonical Pathways		Top Upstream Regulators	
Ingenuity Canonical Pathways	−log (*p*-Value)	Ingenuity Canonical Pathways	−log (*p*-Value)
Integrin-linked kinase signaling	2.38 × 10^−4^	WISP2	1.32 × 10^−8^
Tryptophan degradation X	2.63 × 10^−3^	PDGF BB	5.45 × 10^−8^
Putrescine degradation III	2.63 × 10^−3^	LIMA 1	1.30 × 10^−7^
Dopamine degradation	4.11 × 10^−3^	Estrogen Receptor	2.01 × 10^−7^
Noradrenaline & adrenaline degradation	1.03 × 10^−2^	NTRK-1	3.77 × 10^−7^
